# A feasibility study on exhaled breath analysis using UV spectroscopy to detect COVID-19

**DOI:** 10.1088/1752-7163/ad0646

**Published:** 2023-11-02

**Authors:** Saurin R Sutaria, James D Morris, Zhenzhen Xie, Elizabeth A Cooke, Shavonne M Silvers, Grace A Long, Dawn Balcom, Subathra Marimuthu, Leslie W Parrish, Holly Aliesky, Forest W Arnold, Jiapeng Huang, Xiao-An Fu, Michael H Nantz

**Affiliations:** 1 Departments of Chemistry, University of Louisville, Louisville, KY 40292, United States of America; 2 Chemical Engineering, University of Louisville, Louisville, KY 40292, United States of America; 3 Anesthesiology and Perioperative Medicine, University of Louisville, Louisville, KY 40292, United States of America; 4 Division of Infectious Diseases, Department of Medicine, University of Louisville, Louisville, KY 40292, United States of America

**Keywords:** UV spectroscopy, COVID-19, diagnosis, exhaled breath, VOC, preconcentration

## Abstract

A 23-subject feasibility study is reported to assess how UV absorbance measurements on exhaled breath samples collected from silicon microreactors can be used to detect COVID-19. The silicon microreactor technology chemoselectively preconcentrates exhaled carbonyl volatile organic compounds and subsequent methanol elution provides samples for analysis. The underlying scientific rationale that viral infection will induce an increase in exhaled carbonyls appears to be supported by the results of the feasibility study. The data indicate statistically significant differences in measured UV absorbance values between healthy and symptomatic COVID-19 positive subjects in the wavelength range from 235 nm to 305 nm. Factors such as subject age were noted as potential confounding variables.

## 1. Introduction

Infection by severe acute respiratory syndrome coronavirus 2 (SARS-CoV-2) causes COVID-19, the respiratory disease first reported in December 2019 and responsible for a global pandemic [1]. There have been nearly seven million deaths worldwide since this outbreak began [2]. In addition to the tremendous loss of life, the pandemic also has disrupted many economic, social, and educational facets of society [3]. The global pandemic has put a spotlight on the dire need for an accurate, rapid, and noninvasive test for COVID-19 because of the highly contagious nature and health effects of the disease.

The emergence of exhaled breath analysis as a noninvasive tool for detecting or monitoring diseases [4–6], such as lung [7, 8] or breast cancer [9], suggests that this approach might also be useful for detecting a viral infection. Indeed, recent reports by Ruszkiewicz *et al* [10], Berna *et al* [11], Chen *et al* [12], McCartney *et al* [13], and Sharma *et al* [14] have shown that infection by variants of SARS-CoV-2 can be diagnosed by analysis of select exhaled volatile organic compounds (VOCs) serving as biomarkers of the disease. Furthermore, the US food and drug administration recently approved an emergency use authorization for a test to diagnose COVID-19 based on exhaled VOCs [15]. In each of these cases except the studies by Ruszkiewicz *et al* and Yao *et al*, who used gas chromatography coupled to ion mobility spectrometry (GC-IMS) for analyses, the breath samples were analyzed using gas chromatography in conjunction with mass spectrometry (GC-MS) to detect and measure the various individual organic biomarkers, many of which remain unidentified.

The instruments for chromatographic separation of the VOC constituents of breath and mass spectral measurements in GC-MS approaches to breath analysis are relatively expensive and increase the overall cost of performing the diagnostic test. In considering ways to streamline breath analysis as well as lower the associated expense, we considered spectroscopic approaches in which a collected breath sample would be subjected to a single, rapid measurement using a less expensive instrument. Many spectroscopic techniques have been used for breath analysis [16]. Nuclear magnetic resonance spectroscopy has been used to analyze exhaled breath condensate and was able to differentiate between asthmatic and healthy patients [17]. Selvaraj *et al* have reviewed mid-infrared sensing techniques for exhaled breath diagnostics of a wide range of diseases and detection of potential biomarkers [18]. In a more recent finding, Laird *et al* reported the use of Fourier transform infra-red spectroscopy to detect chemical components in the breath from COVID-19 positive symptomatic and asymptomatic patients and obtained significant results that distinguish patients with or without COVID-19 disease [19]. Iwata *et al* reported the use of UV-Vis spectroscopy to measure the concentration of isoprene in exhaled breath [20]. UV measurements of exhaled breath have an advantage over using infrared (IR) spectroscopy because water has a strong absorption in the mid-infrared spectrum and much less in the UV spectrum. Desiccants are often used with IR spectroscopy approaches to remove exhaled water due to its interference, but the desiccant may also remove potential VOC markers from the exhaled breath in the drying process. A superior water reduction technique, however, recently has been disclosed by Maiti *et al* that improves the use of IR spectroscopy for breath analysis [21].

With these considerations in mind, we explored breath analysis using UV spectroscopy as a means to circumvent the need for chromatographic separation of the individual VOC components in breath. Specifically, we postulated that monitoring the combined absorbance of the highly UV-responsive *α, β*-unsaturated carbonyl compounds present in breath as well as any increases in saturated carbonyls might be a good indicator of net oxidative stress and inflammation, which can be expected to correlate closely with viral infection [22, 23]. There have been many reports linking COVID-19 with oxidative stress and inflammation [24–26]. Oxidative stress correlates closely with an increase in lipid peroxidation-derived aldehyde metabolites in exhaled breath [27, 28]. In agreement with this notion, many of the aforementioned breath analysis studies on COVID-19 patients identified carbonyl biomarkers, including recent studies by Grassin–Delyle *et al* [29] and Liangou *et al* [30], who reported the lipid peroxidation-derived aldehydes nonanal and heptanal as important markers for identification of COVID-19. We report herein on the feasibility of using UV spectroscopy to diagnose COVID-19 from absorbance measurements on the carbonyl compound subset of exhaled VOCs.

### 1.1. Isolation of carbonyl metabolites in exhaled breath

**Figure 1.**
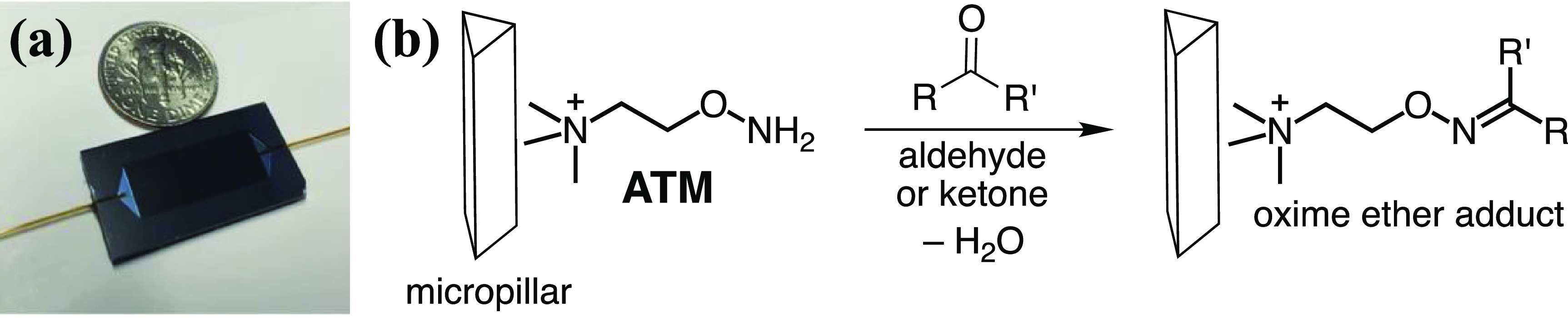
Chemoselective preconcentration of breath carbonyls: (a) silicon micropreconcentrator fitted with inlet and outlet ports relative to a US coin; (b) click chemistry oximation reaction of micropillars coated with ATM to capture carbonyl analytes as non-volatile oxime ether adducts.

We have previously reported a chemoselective preconcentration approach using a silicon microreactor for isolating the carbonyl subset of VOCs in exhaled breath [31–33]. The microreactor contains thousands of micropillars coated with a 2-aminooxy-*N,N,N*-trimethylethan-1-ammonium salt (ATM) [34] for selective concentration of carbonyl VOCs (figure 1). The chemoselectivity of this approach allows for the analysis of carbonyl biomarkers without interference from the many other VOCs in exhaled breath samples, while the preconcentration allows for the accurate measurement of carbonyl biomarkers in the nano- to pico-molar range [35, 36]. ATM-derivatisation also serves the purpose of converting a carbonyl VOC to a charged (cationic) non-volatile salt. After a one-liter breath sample is passed through the microreactor, the ATM-carbonyl adducts are collected from the microreactor by elution with methanol (ca. 200 *µ*l). This preconcentration process accounts for a 5000-fold increase in the concentration in carbonyl VOCs for analysis. In the present work, an aliquot of the eluted solution is transferred to a quartz cuvette for direct measurement using a UV-Vis spectrophotometer.

### 1.2. UV transitions of saturated and α, β-unsaturated carbonyl compounds

**Figure 2.**
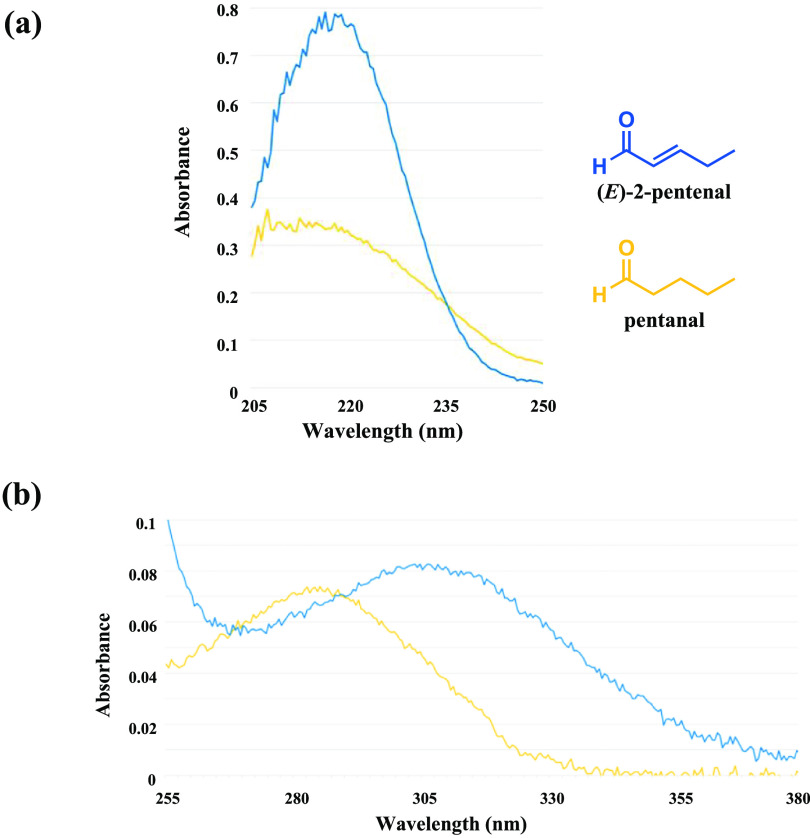
(a) Comparison of pentanal and 2-pentenal *π* → π* UV absorbance spectra. Measurements were obtained from methanol solutions of pentanal and 2-pentenal at concentrations of 22.5 mM and 0.005 mM, respectively. (b) Comparison of pentanal (yellow line) and 2-pentenal (blue line) *n* → π* UV absorbance spectra. Measurements were obtained from methanol solutions of pentanal and 2-pentenal at concentrations of 22.5 mM and 0.1 mM, respectively.

To induce measurable changes in the UV absorption of a breath sample solution due to an increase or decrease in carbonyl biomarkers exhaled by a COVID-19–positive subject relative to a healthy subject, the variable carbonyls should ideally have UV transitions with large molar absorptivities so that even trace-level changes can be expected to register an effect. The relationship between absorbance and molar absorptivity is described by the Beer–Lambert Law [37], represented by the equation *A*= *ϵ*·*l*·c, where *A* is absorbance, *ϵ* is molar absorptivity (sometimes referred to as the molar extinction coefficient, which is directly related to probability of the electronic transition), *l* is length of sample cell, and *c* is concentration of sample solution. Shown in figure 2 are plots of the UV absorption spectra of pentanal (saturated carbonyl) and 2-pentenal (unsaturated carbonyl), serving as representative breath carbonyls, to illustrate how the much larger molar absorptivity of an *α, β*-unsaturated carbonyl compound, which is often more than 10-fold higher than a corresponding transition for the saturated analog, contributes to overall absorption even when present at a trace-level concentration. First, the *λ*_max_ (wavelength at maximum absorption) for each principal electronic transition observed for these compounds differ. The pentanal *π* → π* transition *λ*_max_ is 207.5 nm, whereas the 2-pentenal *π* → π* transition *λ*_max_ is 216.5 nm (figure 2(a)), and the pentanal *n* → π* *λ*_max_ transition is 282.5 nm, whereas the 2-pentenal *n* → π* *λ*_max_ transition occurs at 309 nm (figure 2(b)). The major difference between these two carbonyls is the intensity of their absorbance. With respect to figure 2(a), note that the concentrations of the two carbonyls differ substantially, with the solution of 2-pentenal being 4500-fold less concentrated, yet still absorbing more UV light than the more concentrated solution of pentanal. Although saturated and conjugated unsaturated aldehydes may have *n* → π* molar absorptivities in a similar range, the *π* → π* molar absorptivities of conjugated unsaturated aldehydes tend to be significantly different (hence the greater absorbance difference between figure 2(a) vs. Figure 2(b)). In the case of the *n* → π* transition, the bathochromic shift in absorbance for pentenal due to conjugation results in absorption at wavelengths where the saturated counterpart has no absorption (*λ* > 335 nm, figure 2(b)). In this example, pentanal absorption at 335 nm is near zero whereas 2-pentenal has a measurable absorption at this wavelength, suggesting that screening mixtures of aldehydes for absorptions at these higher wavelengths may be a feasible means of monitoring the appearance of unsaturated carbonyls in a breath sample mixture.

## 2. Materials and methods

All UV-Vis spectra were taken in LC-grade methanol (⩾99.9%, VWR Chemicals BDH) using a VWR Cell Quartz 100 *µ*l Z8.5 mm cuvette and a Beckman Coulter DU 800 spectro-photometer. IR 3-(2-benzothiazolyl)-7-(diethylamino)coumarin was purchased from Chemodex. Tedlar bags with a volume of one liter were purchased from Sigma–Aldrich. Chemical reagents were purchased from Sigma–Aldrich and used without further purification.

### 2.1. Silicon microreactors

The microreactors were fabricated from 4″ silicon wafers using standard microelectromechanical systems fabrication techniques. Details of the microreactor design, fabrication, and characterization have been published elsewhere [32, 33]. The microreactor has thousands of equilateral triangular micropillars (figure 1(b)) in an area that is 21 mm in length and 7 mm in width. The micropillars have a 50 *µ*m lateral length and are 400 *µ*m tall. The distance between the two closest pillars is 10 *µ*m. The micropillar surfaces were oxidized using a wet (mixture of H_2_O and O_2_) thermal oxidation process. The inlet and outlet of the microreactor were fitted with 350 *µ*m O.D. and 250 *µ*m I.D. deactivated fused silica tubes using a silica-based bonding agent. The total empty volume of the microreactor is about 30 *µ*l.

### 2.2. Microreactor coating

The surfaces of the channels and micropillars were functionalized with ATM triflate (ATM·OTf) [38] and an internal reference (IR) 3-(2-benzothiazolyl)-7-(diethylamino)coumarin by infusing the microreactor with a methanol solution (35 *µ*l) containing ATM·OTf (1.75 × 10^–3^ mmol) and IR (3.59 × 10^–8^ mmol) followed by evaporation of the solvent under vacuum overnight at 50 °C.

### 2.3. Institutional review board (IRB)

This research was conducted under protocol 20.1154 approved by the University of Louisville IRB and in accordance, as applicable to the study, with the principles embodied in the Declaration of Helsinki. All participants gave written informed consent to participate in the study. Study subject information can be found in the supplementary material (table S.1).

### 2.4. Collection of exhaled breath samples

Exhaled breath samples were collected from 10 healthy subjects and 13 symptomatic COVID-19 subjects at a University of Louisville Hospital clinic using 1 l Tedlar bags. Nasal swab samples were collected for reverse transcription-polymerase chain reaction to determine COVID-19 infection before collection of exhaled breath samples for all subjects. The Tedlar bag was connected to a Teflon tube to serve as a mouthpiece. Subjects blew through the mouthpiece to fill the 1 l Tedlar bag in one exhaled breath. After collection, the gaseous breath in the Tedlar bag was evacuated through the microreactor at a flow rate of 7 ml min^−1^ (ca. 2 h and 20 min. per evacuation). The setup for the evacuation to capture carbonyl VOCs requires a vacuum pump to pull the breath sample from the Tedlar bag through the ATM/IR-coated microreactor [32, 33]. After the breath sample had been completely processed, the Tedlar bag was discarded as biohazardous waste and the microreactor was eluted with 200 *µ*l methanol. The eluent was used directly for UV-Vis analysis. These last two steps take a combined 10–15 min.

Study subject information is given in table 1. Participants were asked to be at least one hour removed from eating. Liquid intake was not monitored. While some subjects had comorbidities, this information was not collected on all subjects and is a limitation of the present study.

**Table 1.** Study subject information.

	All subjects (*N* = 23)	Positive (*n* = 13)	Negative (*n* = 10)
Age in years (mean)	20–73 (49)	38–73 (59)	20–66 (35)
Gender, male/female	11/12	6/7	5/5
Height in cm	152–183	152–180	156–183
Weight in kg [IQR]	55–113 [23.0]	55–109 [18.0]	63–113 [21.5]
Current smoker	6	5	1

### 2.5. UV analysis

UV-Vis method parameters were as follows: Abs. scan of 200–550 nm, scan speed of 600 nm min^−1^, and a wavelength interval of 0.5 nm. The deuterium and tungsten lamps of the Beckman Coulter DU 800 spectrophotometer were turned on a minimum of thirty minutes before measuring any absorbance data. The sample data were obtained using a VWR Cell Quartz 100 *µ*l Z8.5 mm cuvette. The instrument was reference-blanked using LC-grade methanol. 100 *µ*l of sample was transferred to the quartz cuvette for UV analysis and the cuvette was rinsed with methanol (×3) between samples.

### 2.6. Preparation of ATM-carbonyl standards

Details for synthesis of ATM-pentanal and ATM-pentenal adducts and accompanying characterization data are provided in the supplementary material.

## 3. Results and discussion

### 3.1. Effect of a trace-level α, β-unsaturated aldehyde on UV absorption

We first examined the collective absorption of an ATM–unsaturated carbonyl VOC adduct to determine if concentrations comparable to the trace levels in breath would exceed the limit of detection threshold. While acetone has been reported to have exhaled breath concentrations in the nanomole per liter breath range [39], other saturated and especially *α, β*-unsaturated aldehydes are reported to be in the picomole per liter concentration range [40]. Corradi *et al* searched for potential biomarkers in exhaled breath of non-small cell lung cancer patients and reported 2-hexenal, 2-heptenal and 2-nonenal in exhaled breath at levels ranging from 1.2 to 9.9 picomole per liter breath [40].

**Figure 3.**
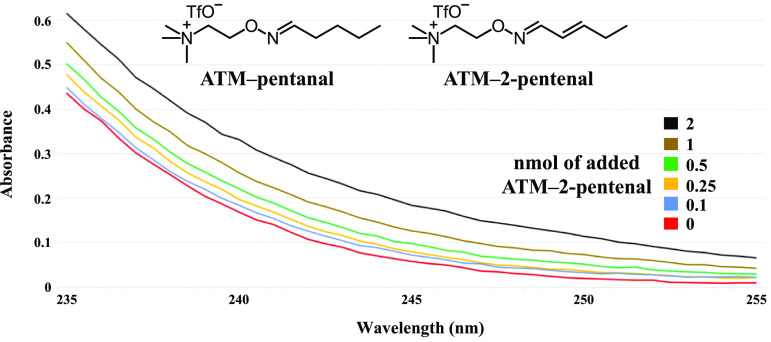
Molecular structures of ATM–pentanal and ATM–2-pentenal and UV absorbance spectra for methanol solutions (200 *µ*l) of ATM–pentanal (50 nmol) with varying amounts of added ATM–2-pentenal (from 0 to 2 nmol).

To understand the effect of an increase in the concentration of an ATM–*α, β*-unsaturated aldehyde adduct on UV absorbance, we measured the UV absorbance of six solutions, each with a constant concentration of a saturated aldehyde adduct and a different concentration of an *α, β*- unsaturated aldehyde adduct (figure 3). For these experiments, two representative ATM–aldehyde adducts were chosen, ATM–pentanal to represent the saturated fraction of aldehydes in breath and ATM–2-pentenal to represent the *α, β*-unsaturated fraction. Each of the six solutions had an ATM–pentanal concentration of 50 nmol/200 *μ*l, which is the post-elution concentration expected from about 1 ppm analyte in 1 l exhaled breath, to represent the larger saturated fraction of exhaled breath aldehydes. The absorbances at wavelengths 235–255 nm (figure 3) showed a near linear relationship between an increase in the *α, β*-unsaturated ATM-2-pentenal adduct concentration in the range of 2.2–44.8 ppb and measured absorbance. Importantly, the absorbance increased due to addition of 0.1 nmol of an *α, β*-unsaturated adduct (representative of an unsaturated, conjugated carbonyl analyte in exhaled breath at 2.2 ppb), suggesting that trace levels of unsaturated carbonyls can induce measurable differences in absorption spectra.

### 3.2. Selection of an IR

**Figure 4.**
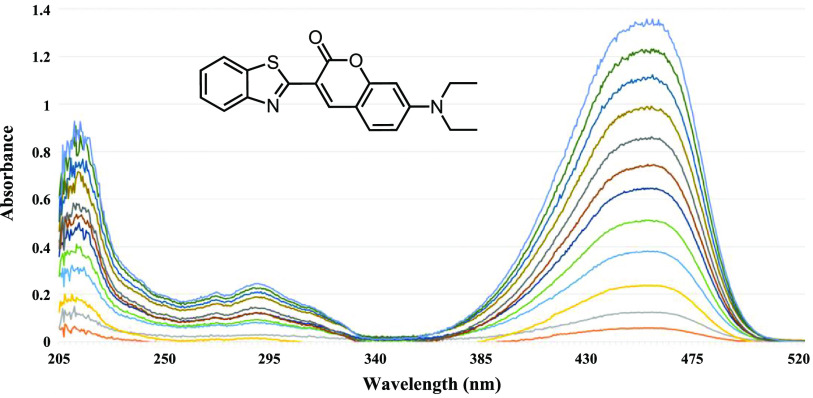
Molecular structure of benzothiazole internal reference and its UV-Vis absorbance spectra in methanol at concentrations from 0.0001, 0.002, 0.004, 0.006, 0.008, 0.01, 0.012, 0.014, 0.016, 0.018, 0.02, to 0.022 mM.

We selected 3-(2-benzothiazolyl)-7-(diethylamino)-coumarin (figure 4) as an IR to monitor proper sample collection and for normalization of absorbance measurements. The thiazole, ester and amino functionality of this IR is not expected to interfere with the oximation chemistry central to capture of carbonyl VOCs within the microreactor. Results of UV-Vis measurements on the IR suggest that if the IR concentration in the volume of eluent typically used to rinse the microreactor (e.g. 200 *µ*l) is in the range of 0.0001 mM, then there will be no interference in the absorbance wavelength range of interest between 235–350 nm yet still provide adequate monitoring capability at its *λ*_max_ of 458 nm (figure 4). The linearity of the IR absorbance response variable at 458 nm was determined separately (*R* ^2^ value = 0.9986; see figure S.1) and found to be well correlated to concentration. We also performed an elution study to confirm that the IR, when co-loaded into the silicon microreactor with ATM and then subjected to the normal drying procedure, can be eluted with methanol from the microreactor. The IR was eluted entirely and structurally unchanged from the microreactor within the first 200 *µ*L aliquot of methanol (see figure S.2).

### 3.3. Feasibility study

Symptomatic COVID-19 positive (13) and healthy volunteer (10) breath samples were taken between 16 June–25 July 2022. According to SARS-CoV-2 variant tracking data from the centers for disease control and prevention, throughout the period that breath samples were taken, 99.9% of subvariants circulating in health and human services region four were the omicron variant [41]. At the beginning of the period the omicron subvariant BA.2.12.1 was dominant, by the end of the period the omicron subvariants BA.4 and BA.5 were dominant [38].

**Figure 5.**
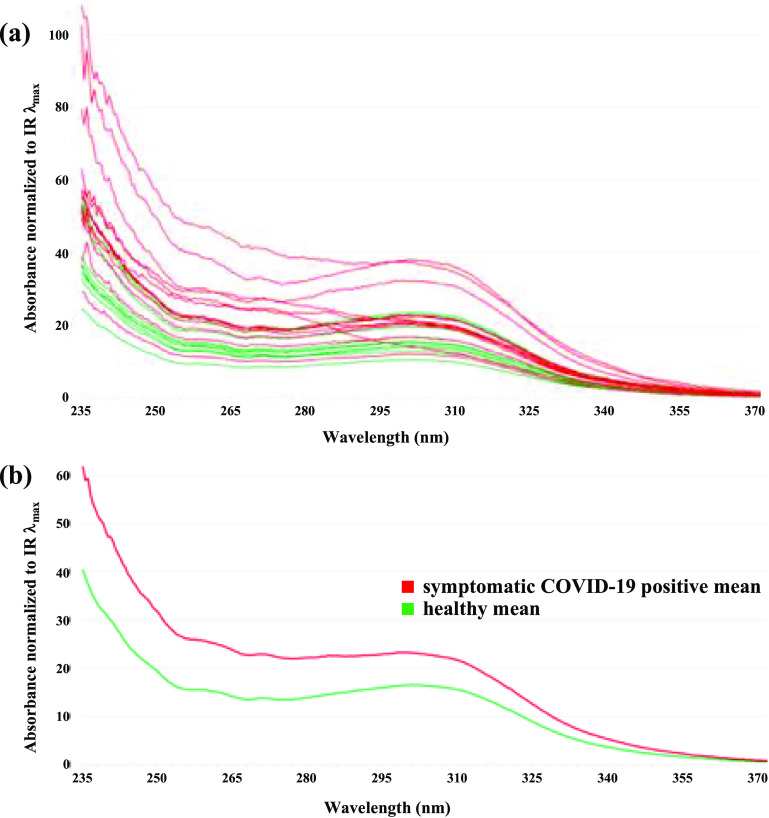
(a) Absorbance spectra of feasibility study samples eluted from ATM/IR-loaded microreactors normalized to the IR absorbance at *λ*_max_ 458 nm. Absorbance of healthy subject samples (10) are shown in green, while symptomatic COVID-19 positive subject samples (13) are shown in red. (b) Mean absorbance spectra of the feasibility study samples.

The UV absorbance values obtained from all subject samples eluted from the microreactors were divided by the absorbance at 458 nm (*λ*_max_ of the IR) from the same spectrum for normalization. The normalized feasibility study data is shown in figure 5(a) where healthy subject data are plotted as green lines and symptomatic COVID-19 positive subject data are the red lines. These preliminary data groupings yield clearly distinct healthy and COVID-19 positive absorbance ranges, which were probed by comparing the mean traces of each group and sorting for the largest differences between means (figure 5(b)). There are many candidate wavelengths to select for prospective studies, from 235 nm to 305 nm, where there is clear separation between the two groups and potential absorbance threshold intensities indicative of COVID-19. One of the largest distinctions between the two means occurs at *λ* 236 nm. A two-tailed Welch’s *t*-test to compare the healthy and COVID-19 positive absorbance means at 236 nm revealed the mean of the normalized absorbance of the 10 healthy patients is 37.3 with a standard deviation of 9.4 and the mean of the normalized absorbance of the 13 COVID-19 positive patients is 59.4 with a standard deviation of 21.1. The difference between the healthy and COVID-19 positive means at 236 nm is statistically significant (*t*(17) = 3.4, *p* < 0.004). Similar *t*-test results were noted at other wavelengths in the 235–305 nm range.

**Figure 6.**
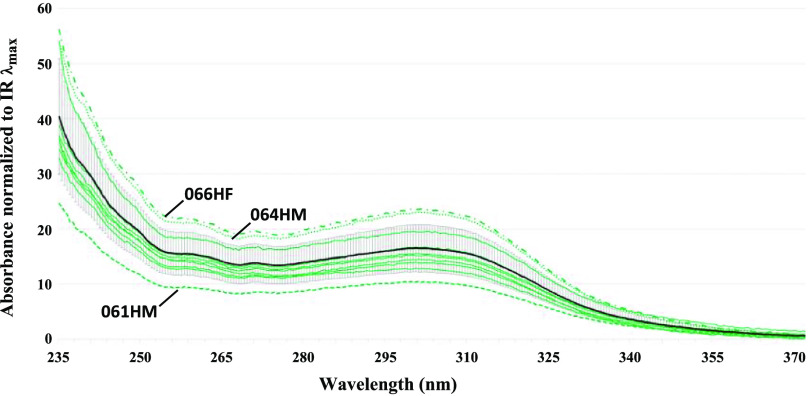
UV absorbance spectra of feasibility study healthy controls and accompanying mean absorbance spectrum (black line) with ±1*σ* error bars. Subject designations are given for the three outliers.

Whereas there is a distinct, measurable increase in net UV absorbance over the wavelength range corresponding to the *π* → π* transition for the carbonyl subset of VOCs exhaled by COVID-19 subjects relative to healthy subjects, some healthy subjects also had elevated carbonyl concentrations. Two healthy subjects had a net UV absorbance greater than one standard deviation from the mean of the healthy absorbances in the 235–305 nm range of interest (figure 6). One healthy subject, 064HM, was a current cigarette smoker. In that smoking cigarettes has been reported to contribute to an increase in exhaled breath carbonyls, in particular formaldehyde and acetaldehyde as well as the *α, β*-unsaturated aldehydes acrolein and crotonaldehyde [42, 43], this result is not surprising [44]. The age of the other healthy subject, 066HF, is sixty-six, which is considerably older than the other healthy subjects in this cohort. Oxidative stress is theorized to increase with age [45, 46]. Interestingly, subject 061HM—the youngest healthy subject of the group at twenty years of age—had a UV absorbance lower than one standard deviation from the healthy subject mean value. Clearly these results point to factors to consider when assessing measurements of exhaled carbonyl VOCs as indicators of infection or disease. To examine the influence of smoking on the COVID-19 positive absorbance data, we compared the absorbance means of COVID-19 positive current-smoker subjects (*n* = 5) vs. COVID-19 positive non-smoker subjects (*n* = 8). In this case, the difference between the COVID-19 positive smoker and non-smoker means at 236 nm is not statistically significant (*t*(4) = 2.2, *p* = 0.09). Another *t*-test, this time excluding current-smoker subject data and using equal variance parameters, comparing the healthy (*n* = 9) and COVID-19 positive (*n* = 8) groups revealed that the difference between absorbance means is still statistically significant (*t*(15) = 3.3, *p* < 0.005).

## 4. Conclusion

Our feasibility study demonstrates that a single UV absorbance measurement of a concentrated breath sample has promise as a screening approach to detect increased levels of carbonyl compounds, which may be indicative of a disease state such as COVID-19. This relatively inexpensive method is enabled by silicon microreactor technology that chemoselectively concentrates exhaled carbonyl compounds as oxime ether adducts. The underlying scientific rationale that the combined UV absorbance of exhaled carbonyl metabolites will increase during a period of viral infection is supported by the results of our feasibility study. Statistically significant UV absorbance differences were noted between healthy and symptomatic COVID-19 positive subjects. The data indicates that lifestyle choices, such as smoking as well as age-related factors, likely should be considered in establishing diagnostic thresholds. Age range-specific absorbance means and thresholds could potentially be established by a clinical study on a much larger sample set to improve accuracy in diagnosing infection. Given the sample size and comorbidity limitations of the present study, a large prospective study that includes asymptomatic subjects is needed to fully assess the potential of this promising new breath analysis approach.

## Data Availability

All data that support the findings of this study are included within the article (and any supplementary files).
